# Multiplex Assay for Protein Profiling and Potency Measurement of German Cockroach Allergen Extracts

**DOI:** 10.1371/journal.pone.0140225

**Published:** 2015-10-07

**Authors:** Taruna Khurana, Ekaterina Dobrovolskaia, Jessica R. Shartouny, Jay E. Slater

**Affiliations:** Laboratory of Immunobiochemistry, Division of Bacterial, Parasitic and Allergenic Products, US Food and Drug Administration, Silver Spring, Maryland, United States of America; Research Center Borstel, GERMANY

## Abstract

**Background:**

German cockroach (GCr) allergens induce IgE responses and may cause asthma. Commercial GCr allergen extracts are variable and existing assays may not be appropriate for determining extract composition and potency.

**Objective:**

Our aim was to develop a multiplex antibody/bead-based assay for assessment of GCr allergen extracts.

**Methods:**

Single chain fragment variable (scFv) antibodies against GCr were obtained by screening libraries derived from naïve human lymphocytes and hyperimmunized chicken splenocytes and bone marrow. Selected clones were sequenced and characterized by immunoblotting. Eighteen scFv antibodies (17 chicken, 1 human) coupled to polystyrene beads were used in this suspension assay; binding of targeted GCr allergens to antibody-coated beads was detected using rabbit antisera against GCr, and against specific allergens rBla g 1, rBla g 2, and rBla g 4. The assay was tested for specificity, accuracy, and precision. Extracts were also compared by IgE competition ELISA.

**Results:**

Chicken scFv’s generated eight different binding patterns to GCr proteins from 14 to 150 kDa molecular weight. Human scFv’s recognized a 100 kDa GCr protein. The multiplex assay was found to be specific and reproducible with intra-assay coefficient of variation (CV) of 2.64% and inter-assay CV of 10.0%. Overall potencies of various GCr extracts were calculated using mean logEC50s for eight selected scFvs. Overall potency measures were also analyzed by assessing the contributions to potency of each target.

**Conclusions:**

An scFv antibody-based multiplex assay has been developed capable of simultaneously measuring different proteins in a complex mixture, and to determine the potencies and compositions of allergen extracts.

## Introduction

Allergen extracts are available in the US as both standardized and non-standardized preparations. Prior to release on the US market, each lot of a standardized allergen extract is compared to a reference standard using a well-defined potency assay. There are 19 FDA-approved standardized allergen extracts; all remaining US-licensed allergen extract are non-standardized extracts for which no potency testing is done [1, 2]. The choice of the best potency assay for a standardized allergen extract depends on the nature and number of relevant allergens. For hymenoptera venom allergen extracts, potency is determined by the mass of dried venom or venom protein in extracts whose integrity is verified using assays for hyaluronidase and phospholipase activity [2]. For allergen extracts in which a single allergen is immunodominant (such as cat and short ragweed pollen allergen extracts) a radial immunodiffusion assay (RID) is used to measure the presence of that allergen (Fel d 1 and Amb a 1, respectively). The potencies of complex allergen extracts, for which no single dominant allergen has been identified (house dust mite and grass pollen allergen extracts) are estimated by competition ELISA using human sera collected from highly allergic subjects [2].

Inhalation of cockroach dust can trigger IgE antibody responses and may induce asthma [3, 4]. Commercially available cockroach extracts are highly variable and contain multiple allergens, some of which have protease activity [5–7]. In addition to the 9 reported allergens of German cockroach (GCr) (www.allergen.org), the following potential allergens have been identified on the basis of IgE binding: vitellogenin, aldolase, Hsp 70, enolase, triosephosphateisomerase, trypsin, chymotrypsin, metalloprotease, and carboxypeptidase [8, 9].

Existing potency assays do not appear to be appropriate for the assessment of GCr allergen extracts. No one or two allergens are immunodominant; hence neither the RID nor any other allergen-specific assay would be appropriate. On the other hand, previous work in our laboratory indicated that competition ELISA may not be able to detect the loss of specific important allergens in a mixed allergen extract [10, 11]. Accurate descriptions of the allergen content of GCr extracts are unlikely to be developed based on existing technologies. Without such characterization, good studies on the efficacy of allergen avoidance measures and allergen immunotherapy will be difficult. Better assessments of these complex reagents may translate into better clinical treatment paradigms.

The goal of the project is to develop an antibody-based multiplex assay for simultaneous measurement of identified and unidentified allergens in GCr extracts and to estimate the overall potency of GCr extracts. This approach would obviate the sometimes challenging decision, early in the standardization of a new product, as to whether the product’s potency is best estimated by measuring its overall IgE binding or the content of specific so-called major allergens, an issue raised recently in a European regulatory document [12]. Earlier our laboratory reported a single chain fragment variable (scFv) antibody-based multiple allergen extract potency assay (MAEPA) for measurement of Fel d 1 and Amb a 1, the major allergens in cat hair and short ragweed pollen allergen extracts respectively [13, 14]. In this report we apply this technique to GCr allergen extracts, and explore its potential as a method to assess the potency and composition of this complex and important allergen source.

## Materials and Methods

### GCr extracts

GCr extracts were prepared from either frozen bodies or defatted whole body GCr powders obtained from commercial source material suppliers. Extracts E2Cg and E3Cg were prepared using ammonium acetate buffer (pH 8.5); extract E2Cg was prepared by Bayer Corp. (Spokane, WA); extract E3Cg was prepared in our laboratory; extracts E4Cg, E6Cg, E7Cg and E8Cg were extracted with PBS [1, 7, 11, 15, 16]. After extraction, filtration and clarification, extracts E2Cg, E3Cg, E4Cg and E6Cg were aliquoted and lyophilized, extracts E7Cg and E8Cg were glycerinated and dispensed into clear glass vials. All of the lyophilized extracts were stored either at 4°C or -20°C. All of the glycerinated extracts were stored at -20°C. GCr extracts were reconstituted in 50% glycerinated H_2_O, stored at -20°C and used within 30 days. Glycerinated commercial extracts A (1:10 w/v), B (1:20 w/v), and C (1:20 w/v) were purchased from three different U.S. allergenic extract manufacturers.

### Generation and purification of scFv antibodies

ScFv antibodies were derived from three sources:

1. Chicken scFv phage library generated against GCr extract as previously described [1].
2. Specific scFv antibodies against Bla g 1, Bla g 2, and Bla g 4 were generated separately. One adult female White Leghorn chicken was immunized with a mixture of recombinant Bla g 1, Bla g 2 and Bla g 4 (Indoor Biotechnologies, VA). Initial dose was 100 μg of each allergen administered with complete Freund’s adjuvant. Boosting doses in incomplete Freund’s adjuvant were administered at 3 and 6 weeks. After 2 months the chicken was euthanized for collection of spleen and bone marrow. The generated phage library was screened against recombinant proteins and high-affinity clones were selected based on their binding to the recombinant Bla g 1, Bla g 2 and Bla g 4 proteins [14].
3. Two different naïve human libraries (complexity level 10^9^) were screened against E6Cg extract to obtain additional anti-GCr scFv antibodies (Creative Dynamics Inc, Shirley, NY and MilleGen, SA LABEGE, Cedex, France).

All of the selected chicken scFv antibodies were cloned into bacterial expression vector pCOMB3X [13]; human scFv clones were expressed in pCDisplay–2 containing 6xHis-tag (Creative Dynamics Inc.). The soluble scFv antibodies were purified from bacterial cell lysates using Ni-affinity chromatography [1, 15].

### Ethics statement

The chicken scFv study was approved by Veterinary Services of Center for Biologics Evaluation and Research, FDA (Animal Research Protocol 2002–48). Chicken was euthanized by CO_2_ asphyxiation in a CO_2_ chamber followed by cervical dislocation.

### Rabbit antisera

Antisera against E6Cg, rBla g 1, rBla g 2, rBla g 3, rBla g 4, and rBla g 7 were raised in New Zealand white rabbits (Spring Valley Laboratories, Woodbine, MD). rBla g 3 was purified as reported earlier [15] and rBla g 7 was purified from soluble fraction of bacterial cell lysate transformed with plasmid pET28b containing Bla g 7 cDNA (plasmid was generously provided by Dr. K.Y Jeong, Department of Internal Medicine, Institute of Allergy, Yonsei University College of Medicine, Seoul, Korea). The rabbits were immunized with aqueous extract (E6Cg) or purified recombinant proteins (rBla g 1 rBla g 2, rBla g 3, rBla g 4, or rBla g 7). Initial dose was 100 μg of each E6Cg or recombinant protein administered with complete Freund’s adjuvant. Two boosting doses (100 μg) in incomplete Freund’s adjuvant were administered at 21 and 60 days. On day 72, animals were euthanized for serum collection and screening by direct ELISA and immunoblotting.

### SDS-PAGE and immunoblotting

Proteins in the extracts were analyzed by SDS-PAGE on 4–12% Bis Tris gels under reducing conditions and visualized with SimplyBlue Safestain (Invitrogen, Carlsbad, CA). For immunoblotting, proteins were transferred to polyvinyl difluoride (PVDF) membranes (pore size 0.2 μm, Invitrogen). After blocking with Starting Block (Invitrogen), membranes were incubated with scFv antibodies (1.0 μg/mL), and binding was detected with HRP-conjugated anti-hemagglutinin (HA) (Roche Diagnostics, Indianapolis) (1:1000 in PBS containing 0.05%Tween 20 and 3% BSA). The blots were developed using chemiluminescent substrate SuperSignal, West Pico (Thermo Scientific).

### Coupling of antibodies to the microspheres

Purified scFv antibodies were coupled to polystyrene carboxy-labeled beads with different spectral properties (Bio-Rad Laboratories, Hercules, CA) [13]. Microbeads were primed with 1-ethyl-3-(3-dimethylaminopropyl)-carbodiimide (EDC) and N-hydroxysulfo-succinimide (sulfo-NHS). Primed beads (2.5x10^6^) were coupled with antibodies (10 μg), washed, counted, and stored in PBS with 0.1% BSA, 0.02% Tween, and 0.05% sodium azide at 4°C in dark.

### GCr Multiplex assay

Immediately before use, the antibody-coupled beads were vortexed and 2x10^3^ beads were dispensed in pre-wet wells of 96-well filter bottom plates (Multiscreen BV; Millipore, Billerica, MA). Assays were run either in monoplex or multiplex format. Antibody-coupled beads were incubated with serially diluted extracts for one hour in the dark at room temperature with gentle mixing. The bound allergens were detected with a mixture of rabbit antisera containing anti-E6Cg, anti-Bla g 1, anti-Bla g 2, and anti-Bla g 4 (each diluted 1:500). Biotin-coupled anti-rabbit IgG antibody (KPL, Gaithersburg, MD) was diluted (1:1000) and added to each well for detection. The binding was detected using diluted streptavidin-R-Phycoerythrin (100 μg/mL) (Thermo Scientific). After the final incubation the beads were resuspended in sheath fluid (Bio-Rad Laboratories) and median fluorescence intensities (MFI) were measured in a BioPlex 200 reader (Bio-Rad Laboratories).

### Thermostability of the scFv binding epitopes

Fifty μL of E6Cg (20 mg/mL) was dispensed into thin-walled microcentrifuge tubes and heated at various temperatures from 40 to 80°C for 2 minutes in heating block wells containing water. After heating, samples were immediately transferred to ice and diluted to 1 mg/mL in PBS containing 1% BSA, for microbead analyses using individual scFv antibodies [15].

### Allergen depletion and detection

Rabbit antisera specific for rBla g 1, rBla g 3, or rBla g 7 were used for depletion of antigens. Normal rabbit serum (Thermo Scientific) was used as a negative control. Protein A agarose bead columns (Thermo Scientific) were washed with PBS before incubating with hyperimmune or normal rabbit sera. Diluted E6Cg extract was incubated with normal rabbit serum beads for 1 hour. The pre-adsorbed extract was then distributed equally into two columns containing protein A beads coupled with either hyperimmune or normal serum and incubated for 2 hours at 4°C. The control and experimental flow-throughs were collected, diluted and analyzed using specific (αBg 1, 2A1, and 6A2) and control (2A1, 6A2 and 6A3) scFv-coupled beads for detection respectively.

### Assay specificity, accuracy, and precision

The specificity of the assay was evaluated by examining commercial allergen extracts from unrelated sources: standardized cat pelt (1:20, Jubilant Hollister-Stier Laboratories, Spokane, WA) standardized short ragweed pollen (1:20, Jubilant Hollister-Stier Laboratories) and *Alternaria alternata* (1:10, Jubilant Hollister-Stier Laboratories). All of the extracts were further diluted 1:10 in PBS containing 1% BSA followed by sequential three-fold dilutions.

The accuracy of the method was determined by performing spiking and recovery studies in monoplex format. GCr extract E6Cg depleted of Bla g 3 was spiked with 2.5 μg/mL and 5.0 μg/mL of rBla g 3. The spiked samples were analyzed in duplicate using scFv 2A1-coated microbeads [15]. A standard curve was generated by diluting rBla g 3 in PBS containing 1% BSA and the percent recovery is reported (observed value/ theoretical value x 100).

Intra-assay precision was calculated as the coefficients of variations (CV) (standard deviation /mean x 100) determined for mean logEC_50_ values for the scFv antibodies analyzed on three independent plates (duplicate wells) on the same day by one operator. Inter-assay precision was assessed by analyzing the extract over a period of three days by one operator (duplicate wells). The reproducibility of the assay was determined by reading MFI values for the assay run on the same day on two different plates and using two different readers.

### Extract Comparison

GCr extracts were analyzed using the multiplex assay for overall potency estimation and individual protein profiling. For each extract, individual antibody-microbead curves were determined and compared. As an estimate of overall extract potency, the logEC_50_ values for each individual antibody-microbead were averaged.

### Statistical Analyses

Multiplex assay data were exported from Bio-Plex Manager to Microsoft Excel 2007 and then into GraphPad Prism 5 (GraphPad Software, La Jolla, CA) for analysis by four-parameter curve fitting. Mean logEC_50_ values were used for comparison among various extracts and non-parametric Mann-Whitney U test was applied to these values for differences among the extracts and for assay variability. CVs were calculated for assay precision.

### Competition ELISA for potency of GCr extracts

Competition ELISA was performed on the GCr allergen extracts as previously described [11] using E2Cg as the reference. E2Cg was used in the assay as the reference because, in preliminary experiments, the dose-responses of E2Cg and the test GCr extracts were comparable. Plates were coated with 10 μg/mL E2Cg. Reference and test extracts were premixed with a reference pooled human-allergic IgE serum (S1Cr) [16]; rabbit anti-E6Cg; or a mixture of eight scFv’s (6A5, 6A2, 2A1, 5D1, 6E1, 6A3, scFv αBg 1 and scFv αBg 4). Detection antibody was either anti-human IgE peroxidase conjugate, goat anti-rabbit IgG peroxidase conjugate, or anti-HA peroxidase conjugate. Relative potency (RP) was determined by comparing the x intercepts of the parallel line inhibition curves [2].

## Results

### Characterization of the scFv antibodies

**Fig 1:**
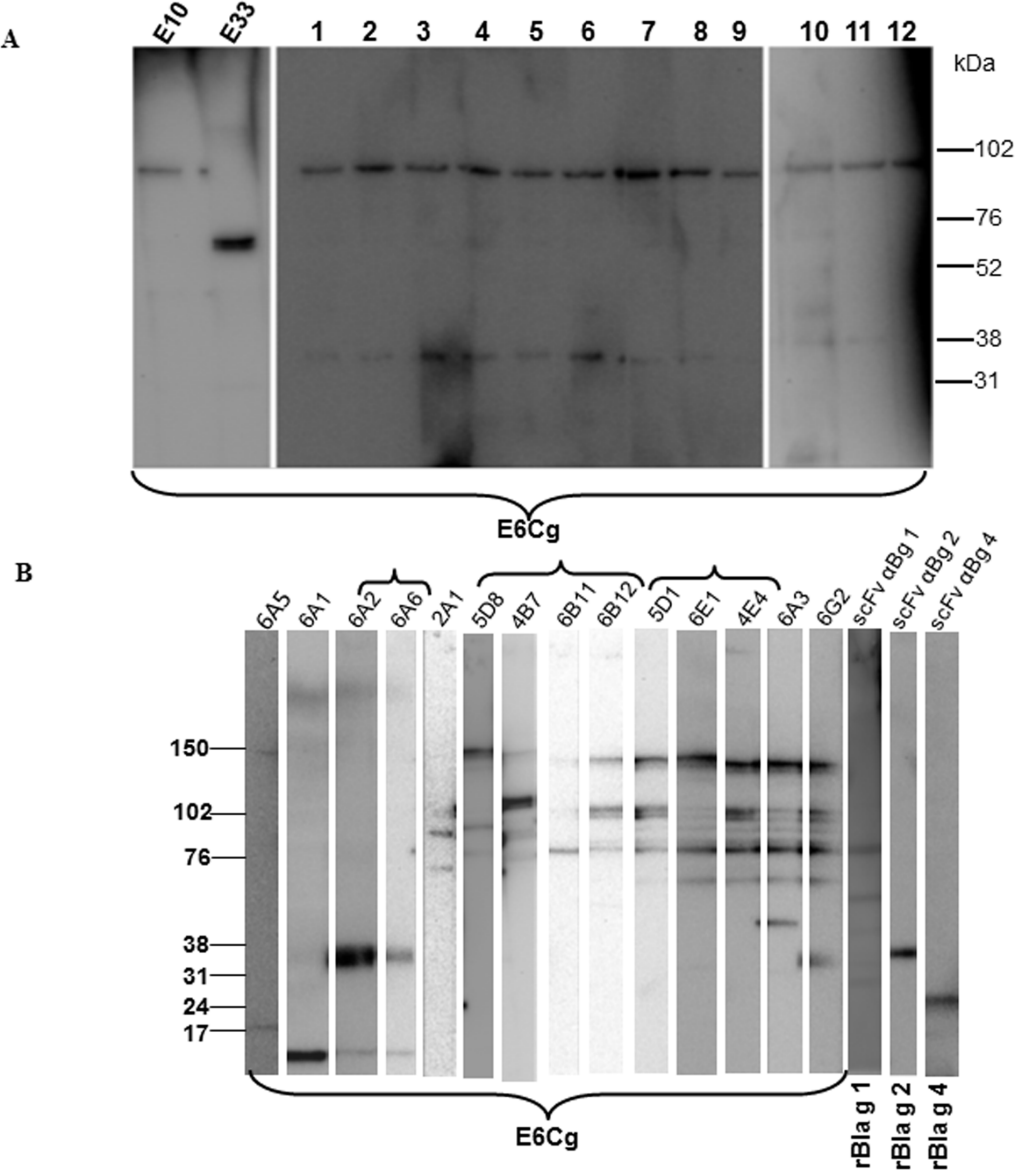
Western blots of E6Cg, rBla g 1, rBla g 2, and rBla g 4. Purified soluble human (A) and chicken (B) scFvs were used as primary antibodies (1.0 μg/mL) and horseradish peroxidase-conjugated anti-hemagglutinin (1:1000) was used for detection using a chemiluminescent substrate. Antibody designations are listed on the top of each panel, and the separated protein sources are indicated at the bottom. Brackets indicate eight different banding patterns.

Human scFv antibodies against E6Cg: After three rounds of biopanning and enrichment of two different naïve human libraries against E6Cg extract, 464 clones were selected for further screening. Following cDNA sequencing (data not shown), 14 unique clones were identified and were further characterized by immunoblotting. The immunoblot screening of the human scFv antibodies revealed that 13 clones bind to a 100 kDa band; some of these also show a very weak signal at 33 kDa molecular mass. One human scFv (E33) recognizes a 70 kDa molecular mass protein (Fig 1A). We were unable to elicit any signal with E33 conjugated microbeads. Therefore, only one of the scFvs that recognized the 100 kDa band (E10) was selected for use in the multiplex assay.

Avian scFv antibodies against E6Cg: The library generated from the hyperimmunized chicken provided 14 unique clones as reported earlier [1]. All of these selected antibodies were purified and tested by immunoblotting for their binding to the proteins present in E6Cg. Eight different binding patterns were observed, with signals ranging from 14 to 150 kDa (Fig 1B). Using mass spectrometry we were able to identify the target of 6A2 as Bla g 7 (not shown) and the target of 2A1 as Bla g 3 [15]. All 14 chicken clones were initially selected for the multiplex assay.

Avian scFv antibodies to specific GCr allergens: None of our scFv antibodies derived from either hyperimmunized chicken or naïve human libraries provided us with scFv antibodies that appeared to recognize the known allergens Bla g 1, Bla g 2, or Bla g 4 (data not shown). Therefore, these scFv antibodies were raised in a specifically hyperimmunized chicken. The selected clones (scFv αBg 1, scFv αBg 2 and scFv αBg 4) were able to recognize recombinant Bla g 1, Bla g 2 and Bla g 4 respectively in an immunoblot (Fig 1B and S1 Table).

### Thermostability of the scFv-binding epitopes

Thermostability of the scFv-binding epitopes was determined by heating E6Cg to 40–80°C for 2 minutes. In general, heat treatment of GCr extract decreased specific scFv binding to target. Two exceptions are antibody 2A1 which recognizes an epitope in Bla g 3 [15] and scFv αBg 4 (S1 Fig).

### Antibody specificity

**Fig 2:**
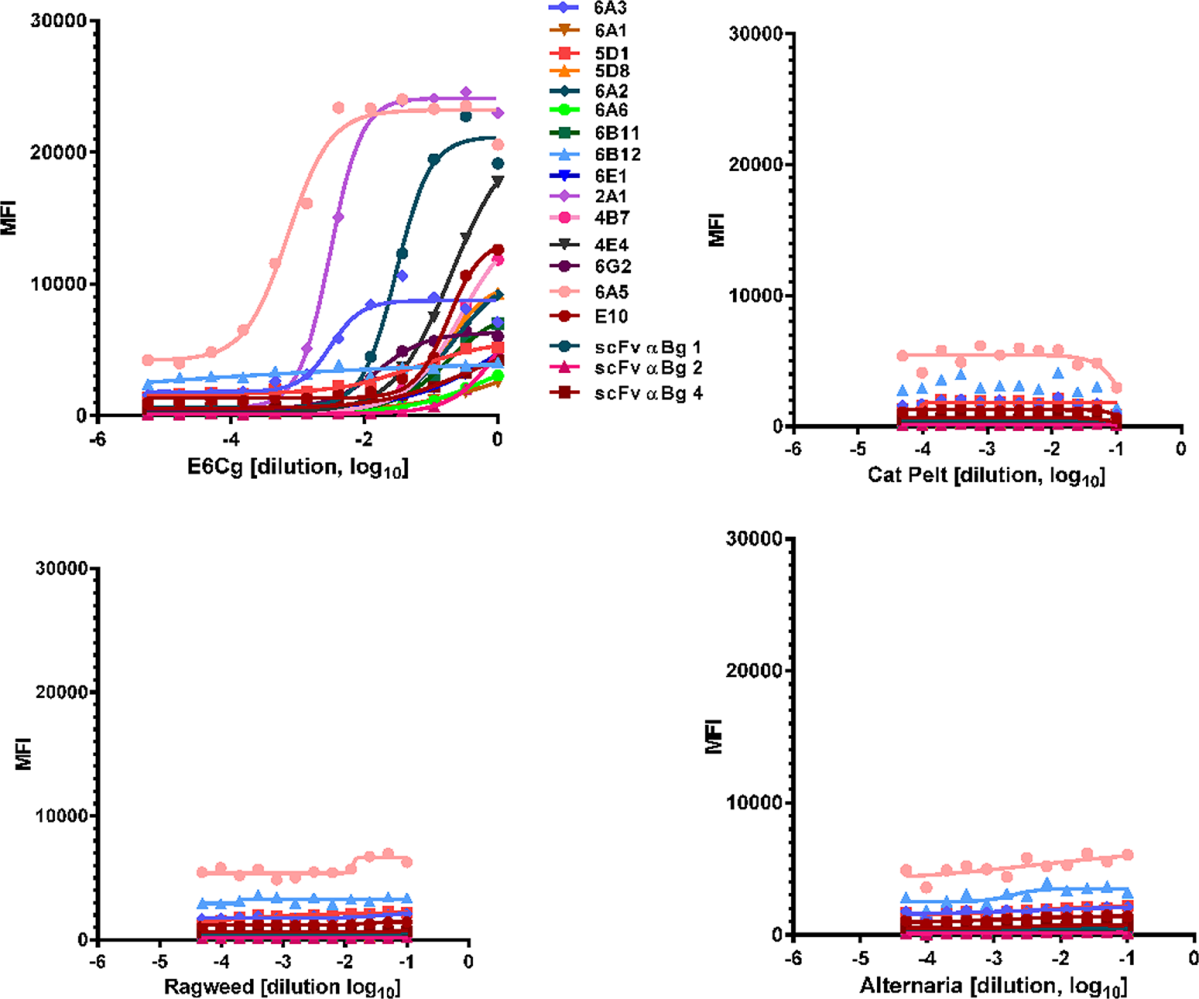
MAEPA specificity. Serially diluted E6Cg (A), cat pelt (B), short ragweed pollen (C) and Alternaria alternata (D) extracts were added to a mix of all 18 scFv-coupled bead sets in 96-well filter bottom plates. Mixture of rabbit anti-E6Cg, anti Bla g 1, anti Bla g 2, and anti Bla g 4 sera (1:500) were used as primary antibody followed by biotinylated anti-rabbit (1:1000) and streptavidin-RPE (1:500). MFI detected for each scFv is on y-axis; extract dilution (log scale) is on x-axis.

The 18 scFv antibodies initially selected for use in the multiplex assay appear to specifically recognize GCr antigens; no signals were noted to any of the three unrelated allergenic extracts chosen (Fig 2A, 2B, 2C and 2D).

**Fig 3:**
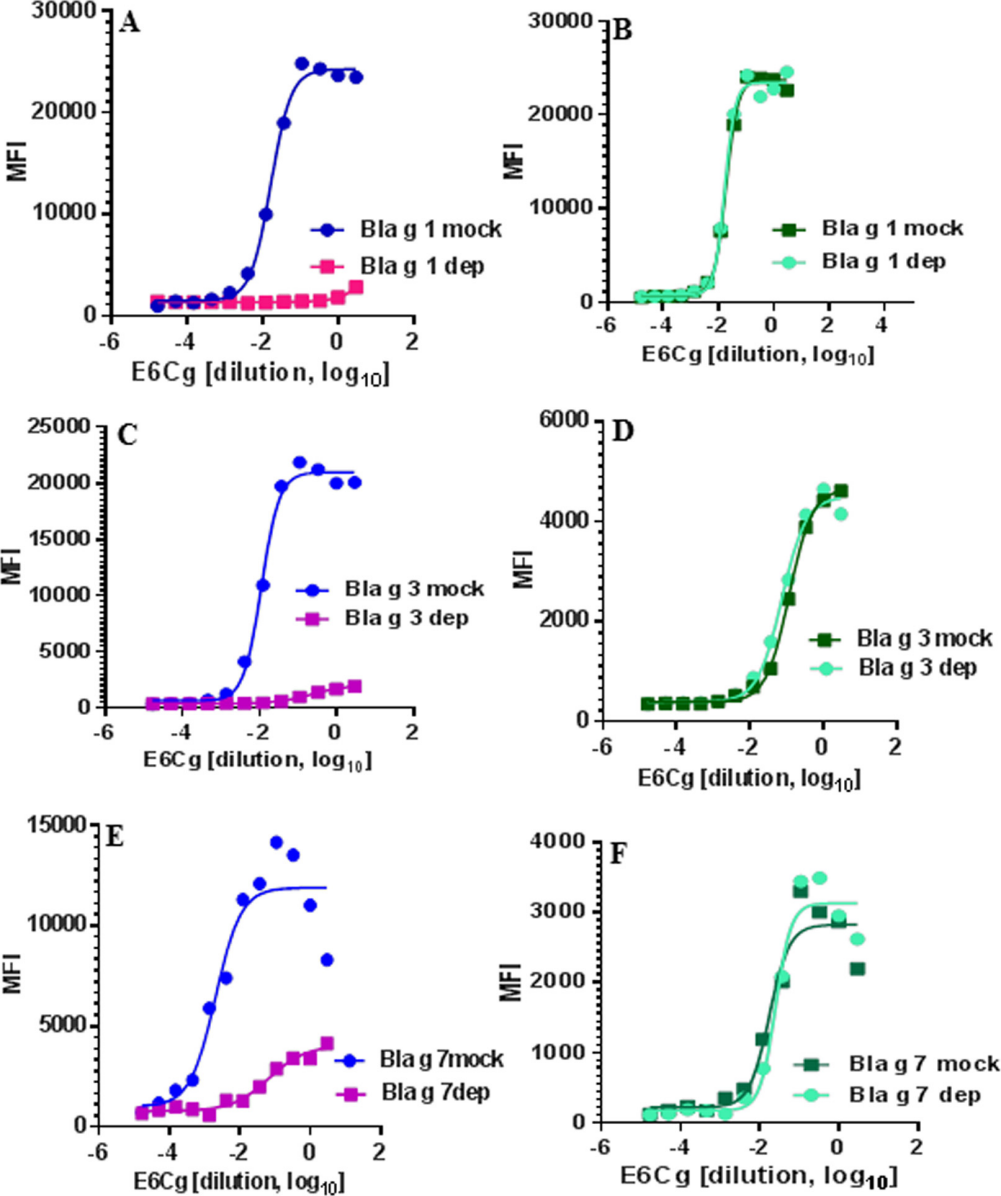
Depletion of E6Cg and detection of specific scFv target proteins. (A) E6Cg depleted of Bla g 1 and mock-depleted analyzed using scFv αBg 1 (C) E6Cg depleted of Bla g 3 and mock-depleted analyzed using scFv 2A1, (E) E6Cg depleted of Bla g 7 and mock-depleted analyzed with scFv 6A2. E6Cg depleted and mock-depleted were also analyzed using non-related scFv antibodies during each run, shown in panels B (scFv 2A1 for detection of Bla g 3), D (scFv 6A2 for detection of Bla g 7), and F (scFv 6A3 for detection of vitellogenin).

Specific binding of selected antibodies to their target proteins was further established by using these antibodies to detect selectively depleted allergens Bla g 1, Bla g 3, and Bla g 7. When specifically depleted E6Cg extracts are tested using microbead-bound antibodies that recognize the depleted targets, the MFI signal is reduced (Fig 3A, 3C and 3E). In contrast, when the same depleted extracts are tested with unrelated microbead-bound antibodies the MFI signal is unchanged (Fig 3B, 3D and 3F).

### Interference among microbead-bound antibodies

LogEC_50_ values were determined when antibody-coupled microbeads were used in monoplex or multiplex format. There is no difference in the mean logEC_50_ between the multiplex and monoplex determinations (S2 Fig) when individual microbead-coupled antibodies pairs are compared (S3 Fig). Minor differences in MFI signals were seen with two antibodies (scFv αBg 1 and 6A1) (S3 Fig).

### Spiking and Recovery

Extracts depleted of Bla g 3 and spiked with different concentrations of nBla g 3 were analyzed using microbead-bound antibody 2A1 [15]. Using the nBla g 3 standard curve (not shown) recoveries of the spiked samples were 83% and 97% at 2.5 μg/mL and 5.0 μg/mL, respectively.

### Precision

The inter- and intra-assay precision of the multiplex assay was determined by comparing logEC_50_ values obtained from three different assays performed on the same day and over a period of three different days. The intra-assay CV was 2.64%; the inter-assay CV was 10%. In addition, no statistically significant differences were observed in logEC_50_ values when two separate plates were read using different readers. The CV was 9.23% (data not shown).

### Evaluation of commercial and laboratory-scale GCr extracts

**Fig 4:**
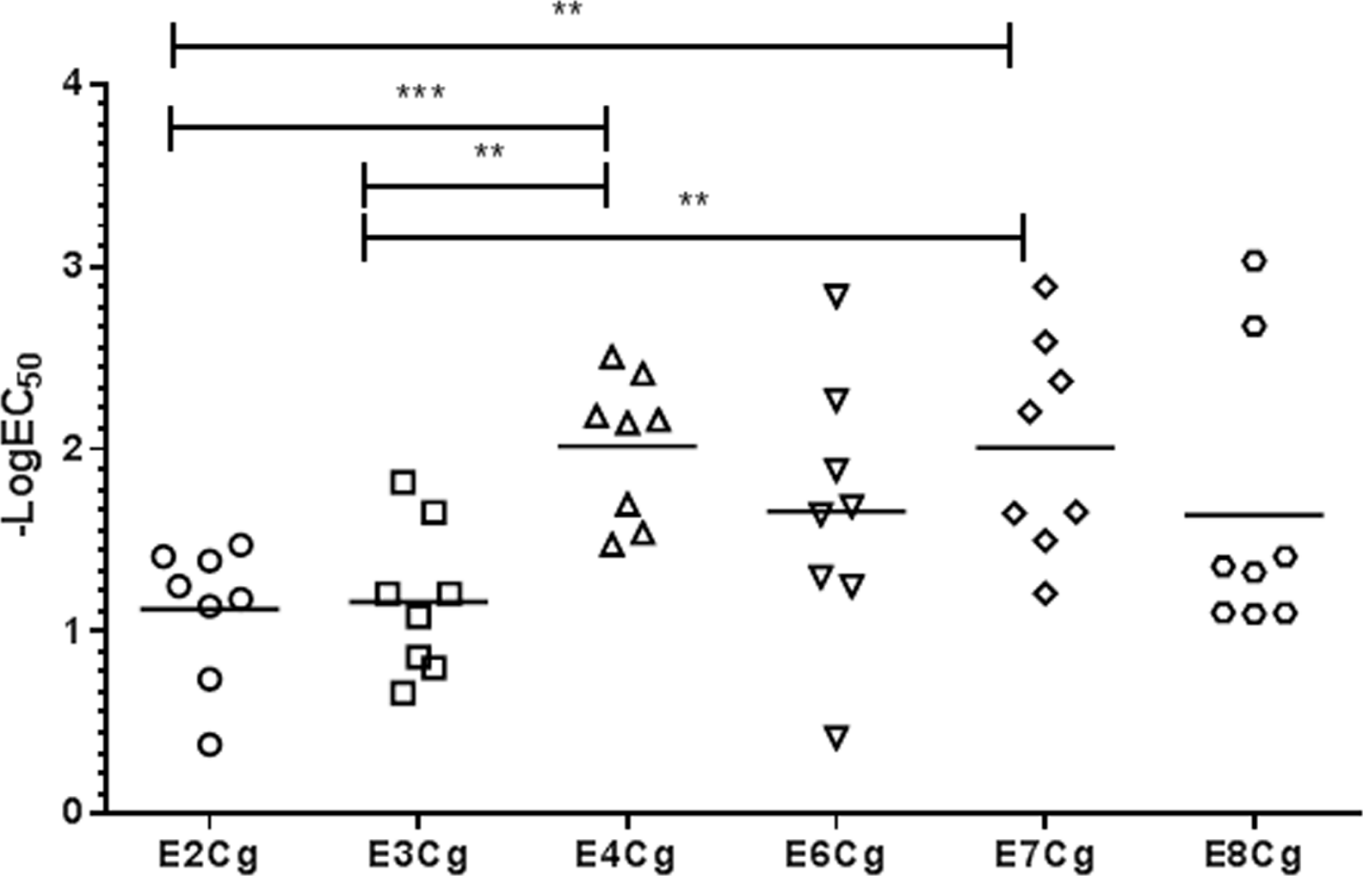
Comparison of laboratory-scale GCr extracts. All scFv-coupled beads for 8 antibodies (see text and S1 Table) were mixed in PBS containing 1% BSA and dispensed in wells containing diluted extract (50 μL/well). Overall mean logEC_50_ values are indicated on y-axis. Each point represents the signal from one scFv-coupled bead; horizontal bar is the mean value. **, p<0.05; ***, p<0.001.

**Fig 5:**
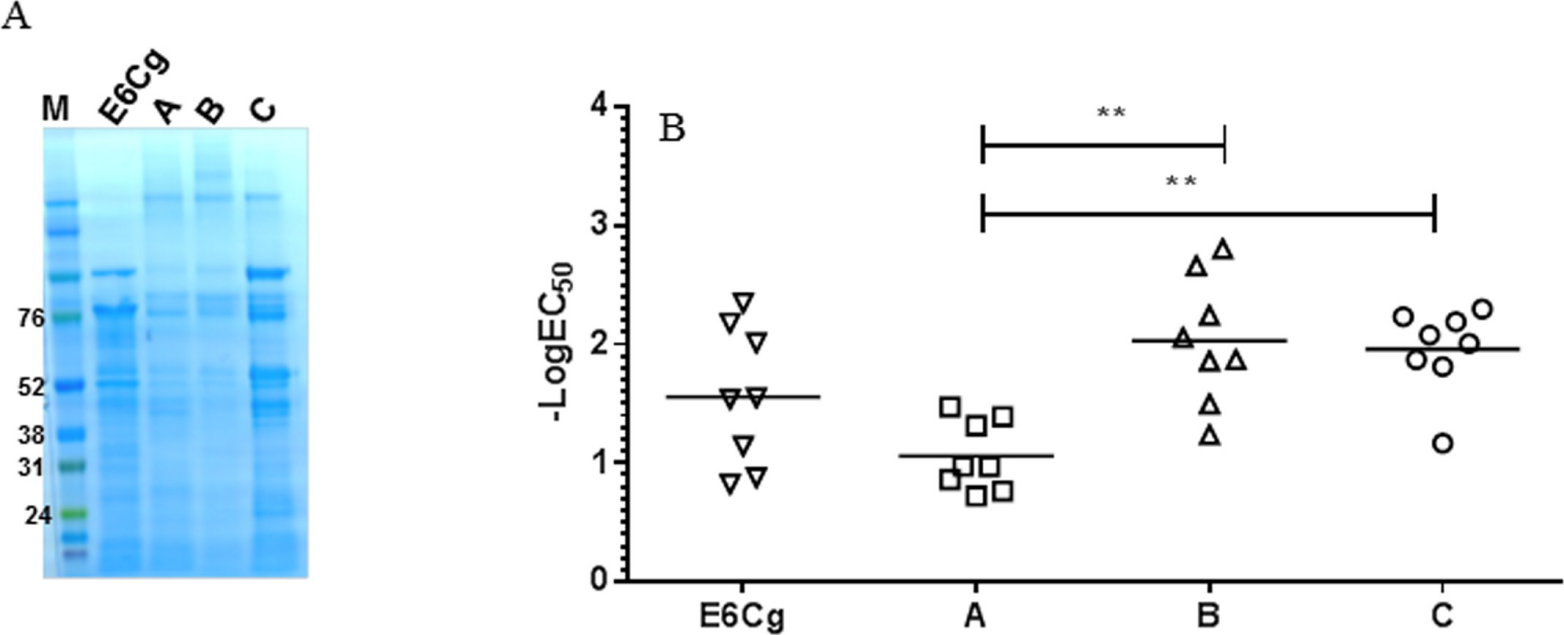
Comparison of GCr extracts from three different manufacturers. (A) SDS-PAGE analysis of GCr extracts was performed under reducing conditions. (B) Overall mean log EC_50_ values are indicated on y-axis. Each point represents the signal from one scFv-coupled bead; horizontal bar is the mean value. **, p<0.05.

We used the multiplex assay to examine potencies and compositions of GCr extracts. To establish an overall potency using this assay, we determined the logEC_50_ for each of 8 scFv’s (6A5, 6A2, 2A1, 5D1, 6E1, 6A3, scFv αBg 1 and scFv αBg 4), and calculated the means of these values. The 8 scFv’s chosen were those that, on repeated testing with various GCr extracts (not shown), yielded consistent and analyzable sigmoidal tracings. Limit of detection of the assay ranges from 5.1x10^-10^ g/L to 1.70x10^-11^ g/L for extract. Using mean logEC_50_ as an estimate of overall potency, extracts E2Cg and E3Cg appear to be equipotent, and less potent than E4Cg and E7Cg (Fig 4); and commercial extracts A, B and C are roughly equipotent to E6Cg, while extracts B and C are more potent than extract A (Fig 5B). SDS-PAGE screening of the commercial extracts was performed for total protein profiling. Overall no difference in migration pattern was noticed among different manufacturers and E6Cg (Fig 5A).

These differences in overall potency were analyzed by assessing the contributions of each of the individual target analytes to the integral signal (S4 and S5 Figs). In general, the signals from the individual analytes correlate with the overall signal.

**Fig 6:**
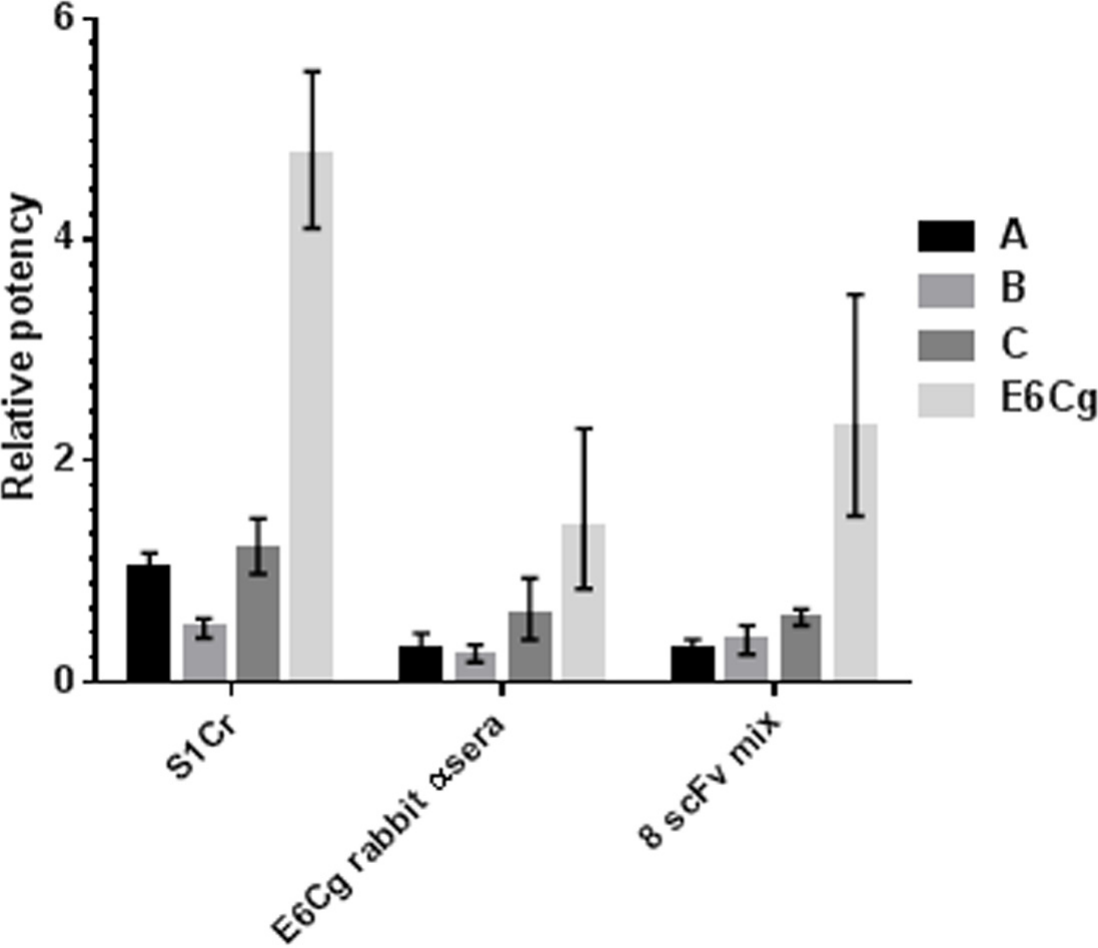
Comparison of GCr extracts from three different manufacturers and E6Cg by competition ELISA using E2Cg as the reference extract. Antibody sources are rabbit anti-E6Cg sera, human serum pool S1Cr, and mix of 8 scFvs. Relative potencies are determined by comparing x intercepts of parallel inhibition curves compared to E2Cg.

When we compared the potencies of the commercial extracts using the multiplex assay to potencies estimated with other, more conventional approaches (competition ELISA using human serum S1Cr, rabbit antiserum, and the 8 scFv’s), the results were discordant with the multiplex analyses. Notably, when determined by competition ELISA using rabbit antiserum or scFv mix, all three commercial extracts were equipotent, but extract B was less potent than extracts A and C when tested with human serum S1Cr. E6Cg was significantly more potent than extracts A, B, and C under all assay conditions (Fig 6).

## Discussion

GCr allergen extracts are non-standardized and, like all non-standardized allergen extracts, are not controlled for the biological potency. Earlier work from this laboratory showed that GCr allergen extracts are highly variable [7]. A recent report suggested that inconsistent results in GCr allergen immunotherapy could be due to insufficient antigenicity of the extract [17]. Standardization of GCr allergen extracts could improve the consistency of these products and may enhance the efficacy of the product for diagnosis and immunotherapy.

Two of the potency assays used for other standardized allergen extracts are RID and competition ELISA. Determination of potency based on measurement of single allergen (RID) does not provide any information about other possible immunopathogenic allergens and conversely, the integrated signal provided by competition ELISA provides no information about the levels of individual allergens [10, 11, 18–20].

We developed MAEPA with the goal of determining the overall potency of GCr allergen extract as well as a profile of multiple component allergens. In developing the specific antibodies for this assay we initially hoped to obtain all necessary antibodies from avian B-cells derived from a GCr-immunized chicken. When we determined that none of the 14 antibodies obtained from this animal were specific for the known GCr allergens Bla g 1, Bla g 2 and Bla g 4, we attempted to obtain additional antibodies from two other sources: another chicken specifically immunized with rBla g 1, rBla g 2, and rBla g 4; and two naïve human libraries screened with the GCr extract E6Cg.

Of the 18 antibodies that we incorporated into the assay, five recognize previously known allergens (Bla g 1, 24–78 kDa; Bla g 2; 37 kDa; Bla g 4, 24 kDa; Bla g 7, 33 kDa; and vitellogenin, 38–150 kDa), and one recognizes a newly described allergen (Bla g 3, 70 kDa), but 12 are directed at proteins of uncertain immunopathogenic significance. This approach was intentional, as we recognized the possibility that not all important GCr allergens have been identified. New allergens not covered by the existing set of 18 antibodies may be incorporated into the assay in the future, and some of the existing antibodies may be removed. Notably, although all 18 scFv antibodies produce a signal when exposed to GCr allergen extracts, only 8 of these produce a quantifiable sigmoidal curve reliably; 4 of these recognize allergens Bla 1, Bla g 3, Bla g 4, and Bla g 7. As such, the MAEPA estimate of overall GCr potency incorporates fewer allergens than we originally might have expected. In particular, the absence of an antibody that detects Bla g 2 is a deficiency that will need to be addressed before using MAEPA in a broader regulatory context.

The role of MAEPA as a regulatory assay for complex allergen extracts remains unclear. In prior work, we demonstrated that MAEPA could be used to estimate the potencies of cat and short ragweed pollen allergen extracts, and that the results replicated determinations using conventional methods [13]. With complex allergen extracts, such as GCr, multiplex assays carry the promise of providing overall potency assessments as well as a profile of specific allergen content. This offers the possibility of a flexible assay providing specific and integrative metrics that can accommodate novel data and information on the best ways to determine allergen potency. Unlike existing approaches to allergen standardization, in which manufacturers decide early in product development between assays that estimate overall potency and those that measure specific individual allergens, assays like MAEPA, if properly developed and validated, can support product potency evaluations both early and late in the product life cycle, without requiring major assay or unitage changes. This could resolve issues expressed by both US [1] and European regulators [21].

In our hands, the GCr MAEPA is specific and reproducible, and we were able to demonstrate specific loss of signal upon depletion of individual components. When stored at 4°C in the dark, the antibody-coupled beads remain stable for 6–12 months (data not shown). Different bead-bound antibodies appear to function in the assay with minimal interference. Our evaluations of existing GCr commercial extracts confirmed their variability [7], and that MAEPA can determine both overall potencies and the differences in individual protein levels among various GCr extracts.

## Supporting Information

S1 Fig Thermostability of E6Cg. Extract was heated at 40°C to 80°C for 2 minutes and cooled rapidly on ice. Heated and unheated extracts were incubated with scFv-coupled bead sets for analysis. The experiment was performed three times with two experimental replicates and MFI is represented as mean ±SD. UnH, unheated extract.
(TIF)

S2 Fig Comparison of monoplex assay and multiplex assays using E6Cg. Duplicate wells were used during experiments. The dose response curves were analyzed using non-linear regression analyses and mean log EC_50_ values were used for t-test analysis.
(TIF)

S3 Fig Comparative analysis of individual scFvs during monoplex and multiplex execution of the assay. Each data point is mean of n = 3 for monoplex and n = 4 for multiplex assay. Error bars are 95% confidence intervals.
(TIF)

S4 Fig Comparative analysis of individual scFvs in multiplex assay used for screening of various laboratory prepared GCr extracts. For clarity error bars are not included.
(TIF)

S5 Fig Comparative analysis of commercial extracts. Multiplex assay used for screening of various commercially available GCr extracts. A, B, and C are three U.S. allergen extract manufacturers. All scFv-coupled beads for 8 antibodies were mixed in PBS containing 1% BSA and dispensed in wells containing diluted extract (50 μL/well). For clarity error bars are not included.
(TIF)

S1 Table Sources of all 18 scFvs antibodies are indicated. Out of 8 scFvs selected for final multiplex assay targets for five are known and included.
(DOC)

## References

[1] Khurana T, Slater JE. Update on the FDA/CBER allergen standardization program. Arb Paul Ehrlich Inst Bundesinstitut Impfstoffe Biomed Arzneim Langen Hess. 2011; 97:37–44. 24912311

[2] Rabin RL, Slater JE. Standardized Allergen Vaccines in the United States In: Lockey RF, Ledford DK, editors. Allergens and allergen immunotherapy. CRC Press, 2014 pp. 281–287.

[3] Morgan WJ, Crain EF, Gruchalla RS, O’Connor GT, Kattan M, Evans R et al Results of a home-based environmental intervention among urban children with asthma. N Engl J Med 2004; 351:1068–1080. 15356304 10.1056/NEJMoa032097

[4] Rosenstreich DL, Eggleston P, Kattan M, Baker D, Slavin RG, Gergen P et al The role of cockroach allergy and exposure to cockroach allergen in causing morbidity among inner-city children with asthma. N Engl J Med 1997; 336:1356–1363. 9134876 10.1056/NEJM199705083361904

[5] Slater JE. Characterization of allergen extracts. Dev Biol 2005; 122:145–152. 16375259

[6] Londres MI, Sarinho FW, Miranda PJ, Sole D, Sarinho E. Allergy to cockroaches: challenges in diagnosis. Clin Lab 2011; 57:969–974. 22239029

[7] Patterson ML, Slater JE. Characterization and comparison of commercially available German and American cockroach allergen extracts. Clin Exp Allergy 2002; 32:721–727. 11994096 10.1046/j.1365-2222.2002.01397.x

[8] Chuang JG, Su SN, Chiang BL, Lee HJ, Chow LP. Proteome mining for novel IgE-binding proteins from the German cockroach (Blattella germanica) and allergen profiling of patients. Proteomics 2010; 10:3854–3867. 10.1002/pmic.201000348 20960453

[9] Jeong KY, Kim CR, Park J, Han IS, Park JW, Yong TS. Identification of novel allergenic components from German cockroach fecal extract by a proteomic approach. Int Arch Allergy Immunol 2013; 161:315–324. 10.1159/000347034 23689614

[10] Soldatova LN, Paupore EJ, Burk SH, Pastor RW, Slater JE. The stability of house dust mite allergens in glycerinated extracts. J Allergy Clin Immunol 2000; 105:482–488. 10719297 10.1067/mai.2000.104549

[11] Dobrovolskaia E, Gam A, Slater JE. Competition enzyme-linked immunosorbant assay (ELISA) can be a sensitive method for the specific detection of small quantities of allergen in a complex mixture. Clin Exp Allergy 2006; 36:525–530. 16630159 10.1111/j.1365-2222.2006.02466.x

[12] Committee for Medicinal Products for Human Use. Guideline on Allergen Products: Production and Quality Issues. European Medicines Agency. 2008. Available: http://www.ema.europa.eu/docs/en_GB/document_library/Scientific_guideline/2009/09/WC500003333.pdf.

[13] deVore NC, Huynh S, Dobrovolskaia EN, Slater JE. Multiplex microbead measurements for the characterization of cat and ragweed allergen extracts. Ann Allergy Asthma Immunol 2010; 105:351–358. 10.1016/j.anai.2010.09.026 21055660

[14] Finlay WJ, deVore NC, Dobrovolskaia EN, Gam A, Goodyear CS, Slater JE. Exploiting the avian immunoglobulin system to simplify the generation of recombinant antibodies to allergenic proteins. Clin Exp Allergy 2005; 35:1040–1048. 16120086 10.1111/j.1365-2222.2005.02307.x

[15] Khurana T, Collison M, Chew FT, Slater JE. Bla g 3: a novel allergen of German cockroach identified using cockroach-specific avian single-chain variable fragment antibody. Ann Allergy Asthma Immunol 2014; 112:140–145. 10.1016/j.anai.2013.11.007 24468254

[16] Patterson ML, Slater JE. Characterization and comparison of commercially available German and American cockroach allergen extracts. Clin Exp Allergy 2002; 32:721–727. 11994096 10.1046/j.1365-2222.2002.01397.x

[17] Wood RA, Togias A, Wildfire J, Visness CM, Matsui EC, Gruchalla R et al Development of cockroach immunotherapy by the Inner-City Asthma Consortium. J Allergy Clin Immunol 2014; 133:846–852. 10.1016/j.jaci.2013.08.047 24184147 PMC3943647

[18] Morrow KS, Slater JE. Regulatory aspects of allergen vaccines in the US. Clin Rev Allergy Immunol 2001; 21:141–152. 11725602 10.1385/CRIAI:21:2-3:141

[19] Slater JE, James R, Pongracic JA, Liu AH, Sarpong S, Sampson HA et al Biological potency of German cockroach allergen extracts determined in an inner city population. Clin Exp Allergy 2007; 37:1033–1039. 17581196 10.1111/j.1365-2222.2007.02751.x

[20] Pomes A, Arruda LK. Investigating cockroach allergens: Aiming to improve diagnosis and treatment of cockroach allergic patients. Methods 2014; 66:75–85. 10.1016/j.ymeth.2013.07.036 23916425 PMC3909726

[21] Lorenz AR, Luttkopf D, Seitz R, Vieths S. The regulatory system in Europe with special emphasis on allergen products. Int Arch Allergy Immunol 2008; 147: 263–275. 10.1159/000146074 18648190

