# Supplementary figures and images for: Multiplex Assay for Protein Profiling and Potency Measurement of German Cockroach Allergen Extracts
Source: PLoS One. 2015 Oct 7;10(10):e0140225. doi: 10.1371/journal.pone.0140225 (PMC4596881; doi:10.1371/journal.pone.0140225)

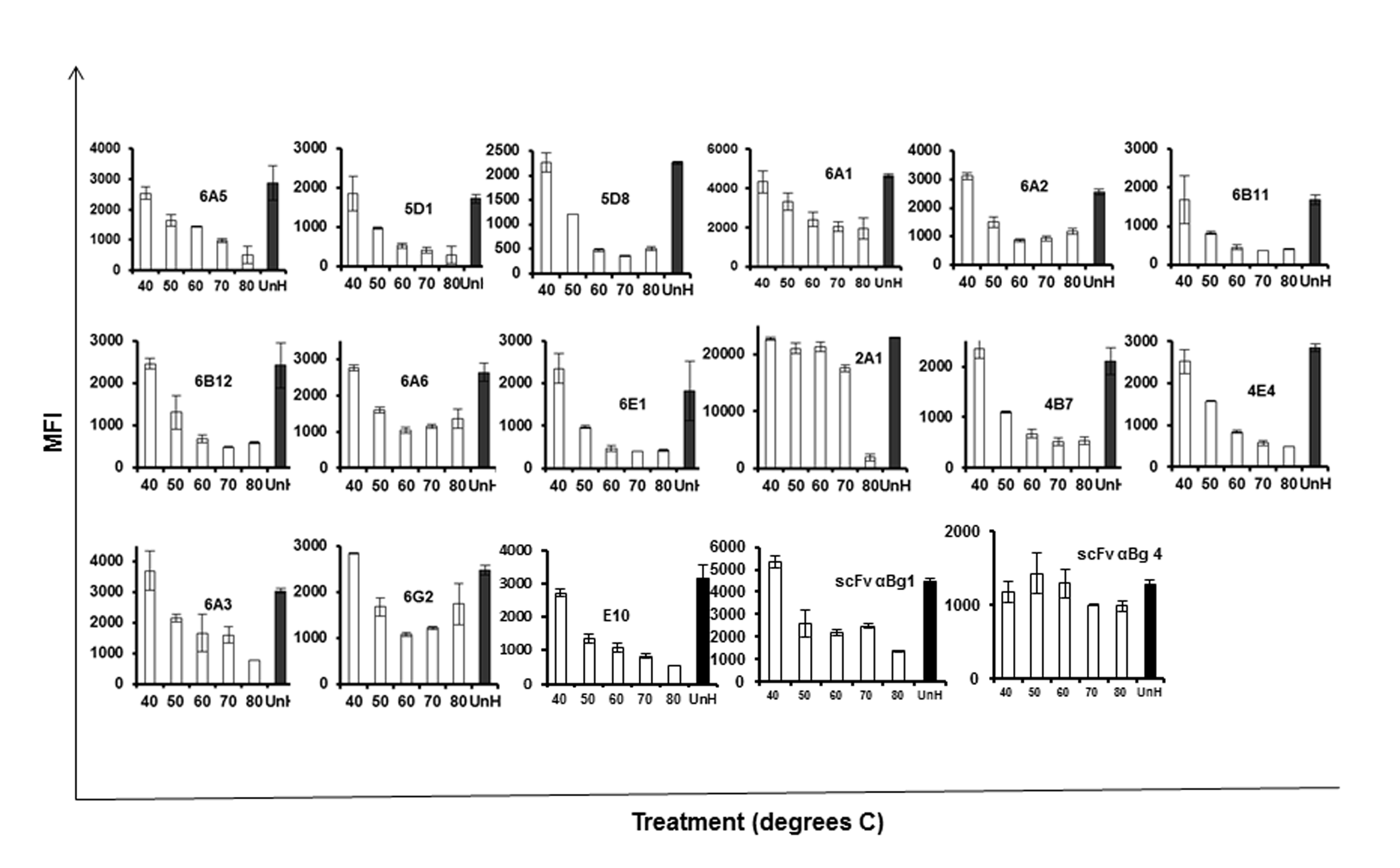

Supplement: S1 Fig — Extract was heated at 40°C to 80°C for 2 minutes and cooled rapidly on ice. Heated and unheated extracts were incubated with scFv-coupled bead sets for analysis. The experiment was performed three times with two experimental replicates and MFI is represented as mean ±SD. UnH, unheated extract. (TIF) [file pone.0140225.s001.TIF]

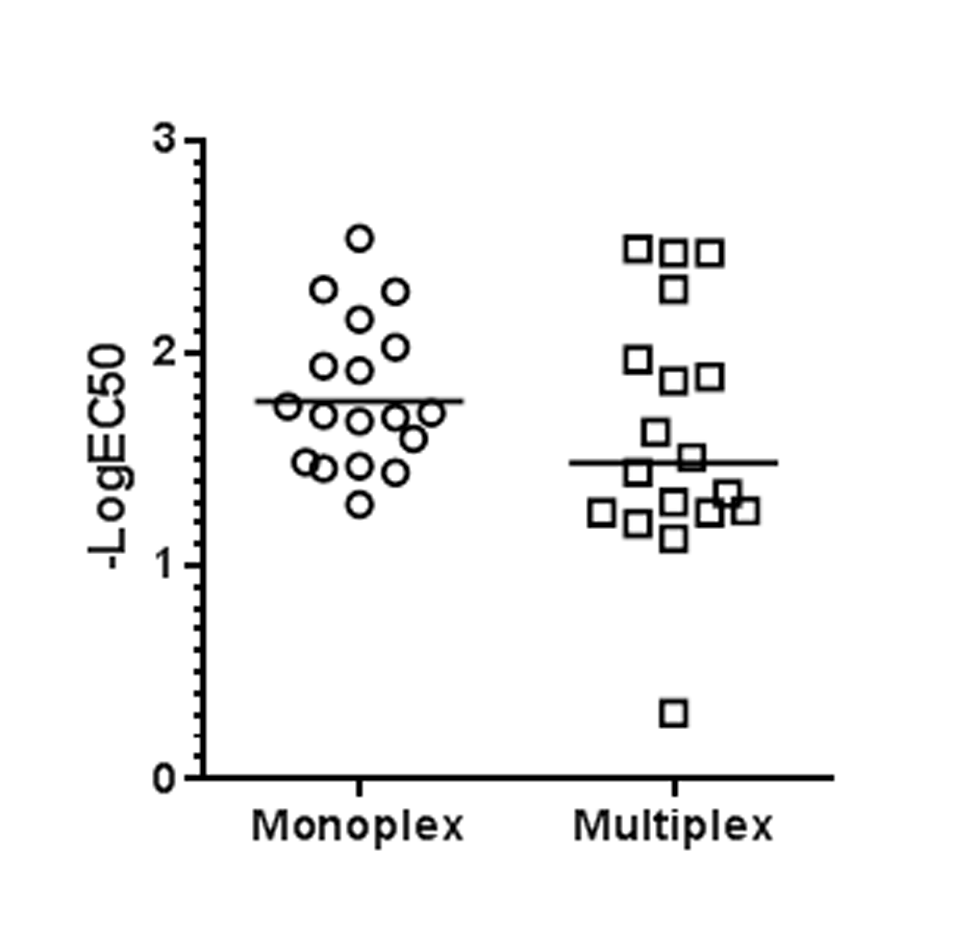

Supplement: S2 Fig — Duplicate wells were used during experiments. The dose response curves were analyzed using non-linear regression analyses and mean log EC50 values were used for t-test analysis. (TIF) [file pone.0140225.s002.TIF]

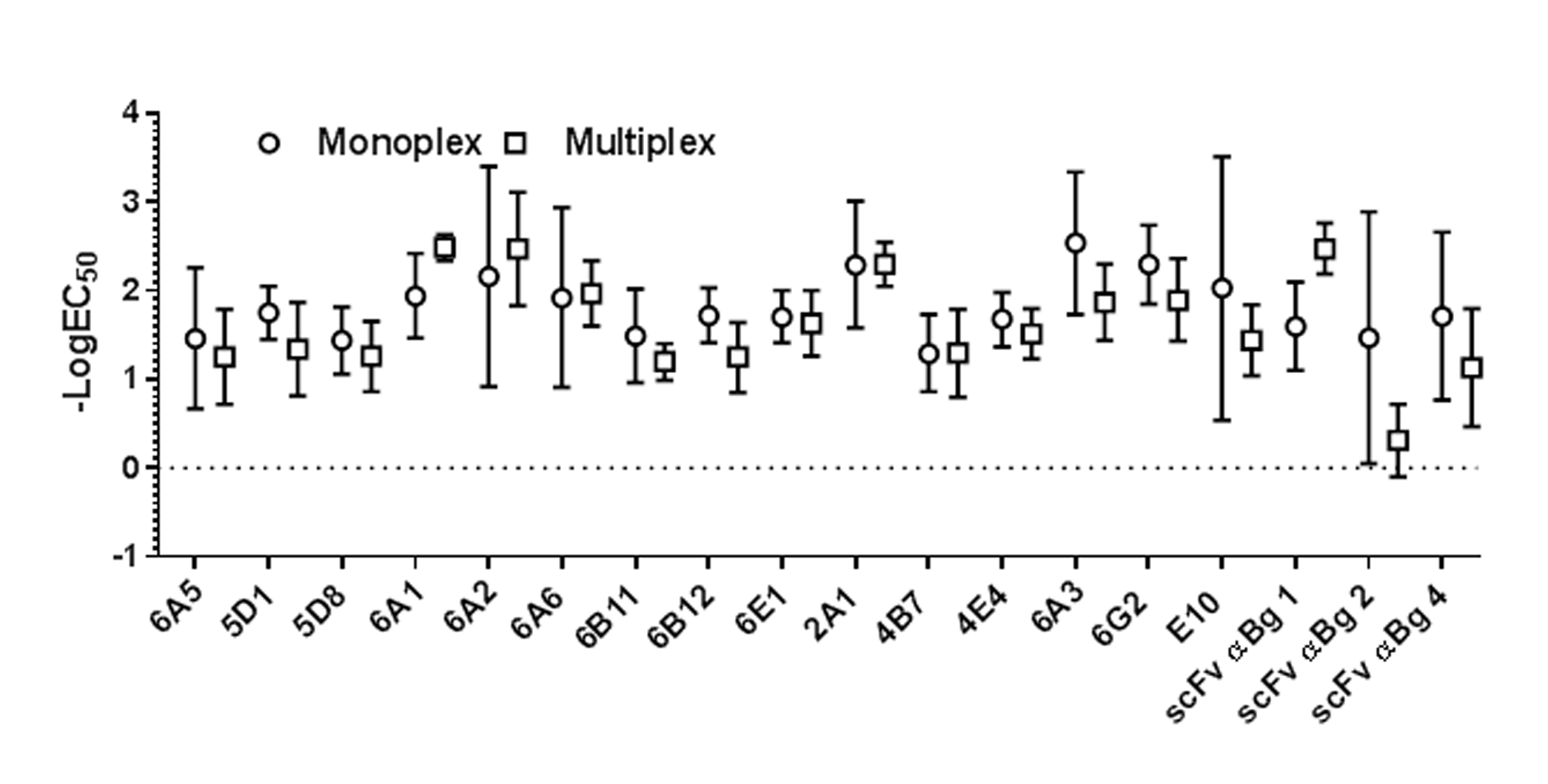

Supplement: S3 Fig — Each data point is mean of n = 3 for monoplex and n = 4 for multiplex assay. Error bars are 95% confidence intervals. (TIF) [file pone.0140225.s003.TIF]

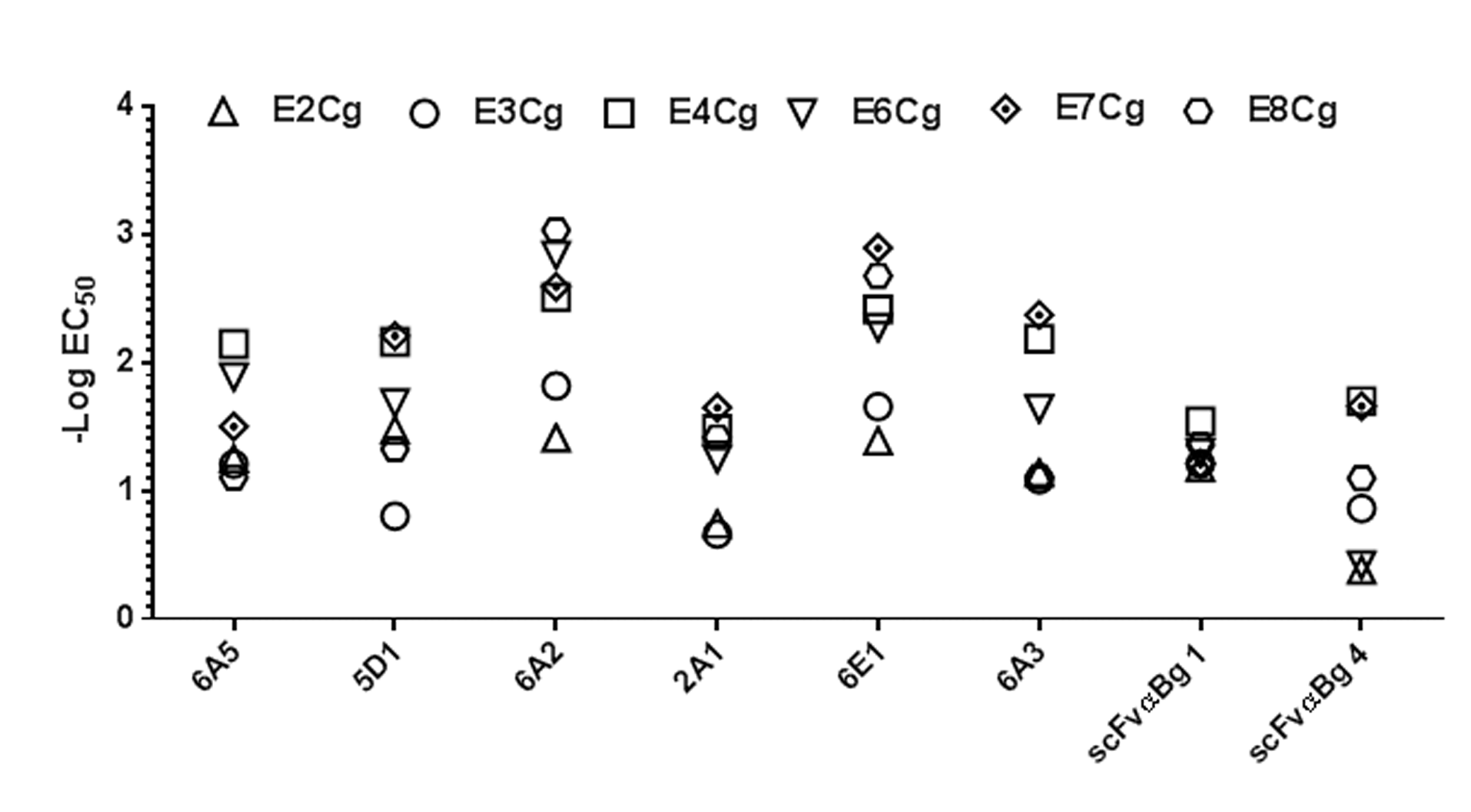

Supplement: S4 Fig — For clarity error bars are not included. (TIF) [file pone.0140225.s004.TIF]

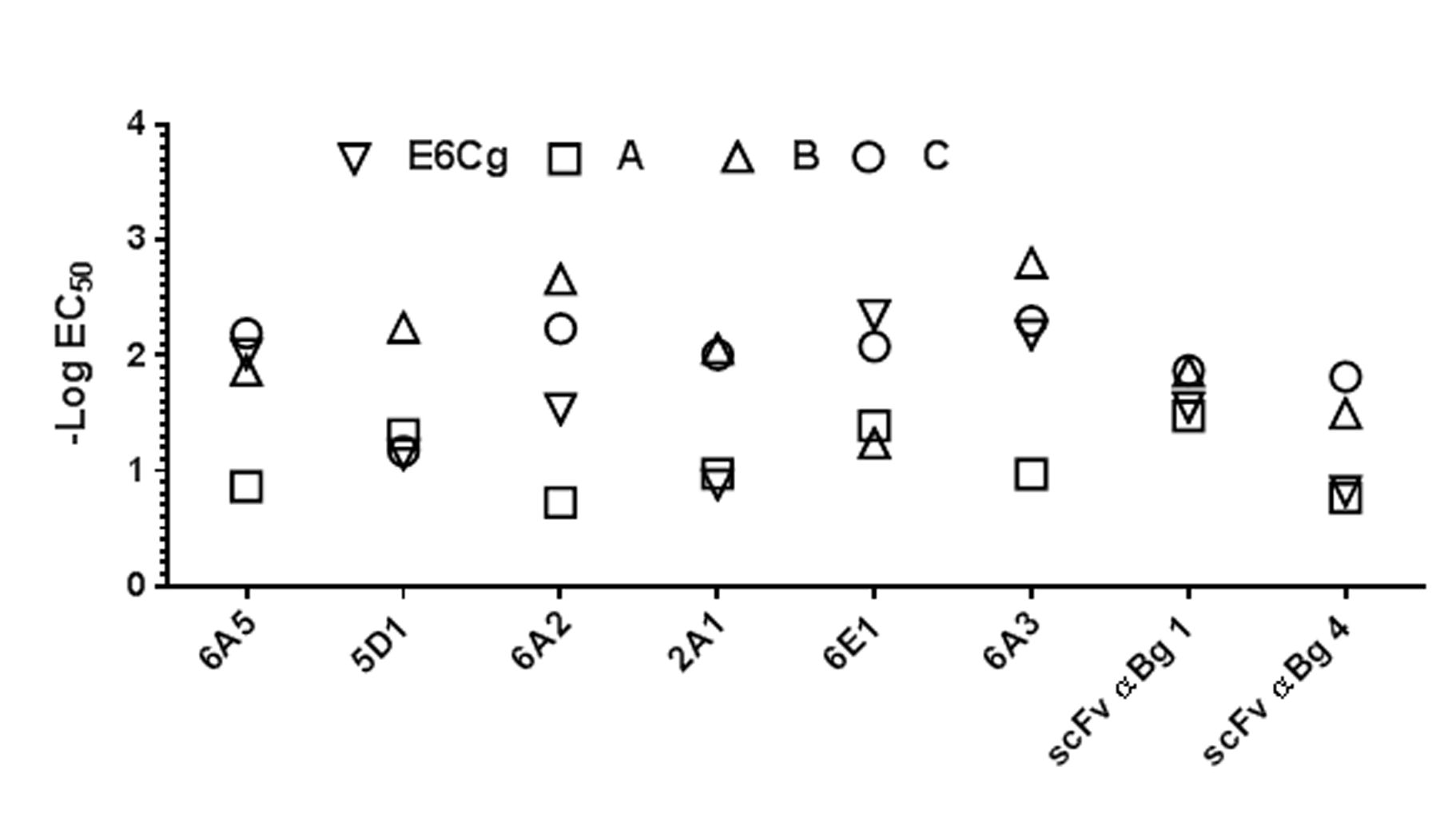

Supplement: S5 Fig — Multiplex assay used for screening of various commercially available GCr extracts. A, B, and C are three U.S. allergen extract manufacturers. All scFv-coupled beads for 8 antibodies were mixed in PBS containing 1% BSA and dispensed in wells containing diluted extract (50 μL/well). For clarity error bars are not included. (TIF) [file pone.0140225.s005.TIF]
