# Supplementary material for: Multiplex Assay for Protein Profiling and Potency Measurement of German Cockroach Allergen Extracts
Source: PLoS One. 2015 Oct 7;10(10):e0140225. doi: 10.1371/journal.pone.0140225 (PMC4596881; doi:10.1371/journal.pone.0140225)
Supplement: S1 Table — Out of 8 scFvs selected for final multiplex assay targets for five are known and included. (DOC) [file pone.0140225.s006.doc]

**Table S1. Sources and target of scFv antibodies**
